# Cognitive behavioral therapy and mindfulness-based cognitive therapy for depressive symptoms in patients with diabetes: design of a randomized controlled trial

**DOI:** 10.1186/2050-7283-1-17

**Published:** 2013-10-09

**Authors:** K Annika Tovote, Joke Fleer, Evelien Snippe, Irina V Bas, Thera P Links, Paul MG Emmelkamp, Robbert Sanderman, Maya J Schroevers

**Affiliations:** Department of Health Sciences, Section Health Psychology, University of Groningen and University Medical Center Groningen, Groningen, the Netherlands; Department of Clinical Psychology, University of Amsterdam, Amsterdam, the Netherlands; Department of Endocrinology, University of Groningen and University Medical Center Groningen, Groningen, the Netherlands; The Center for Social and Humanities Research, King AbdulAziz University, Jeddah, Saudi Arabia

**Keywords:** Cognitive behavioral therapy, Mindfulness, Diabetes, Depression, Treatment, Intervention, Randomized controlled trial

## Abstract

**Background:**

Depressive symptoms are a common problem in patients with diabetes, laying an additional burden on both the patients and the health care system. Patients suffering from these symptoms rarely receive adequate evidence-based psychological help as part of routine clinical care. Offering brief evidence-based treatments aimed at alleviating depressive symptoms could improve patients’ medical and psychological outcomes. However, well-designed trials focusing on the effectiveness of psychological treatments for depressive symptoms in patients with diabetes are scarce. The Mood Enhancement Therapy Intervention Study (METIS) tests the effectiveness of two treatment protocols in patients with diabetes. Individually administered Cognitive Behavioral Therapy (CBT) and Mindfulness-Based Cognitive Therapy (MBCT) are compared with a waiting list control condition in terms of their effectiveness in reducing the severity of depressive symptoms. Furthermore, we explore several potential moderators and mediators of change underlying treatment effectiveness, as well as the role of common factors and treatment integrity.

**Methods/design:**

The METIS trial has a randomized controlled design with three arms, comparing CBT and MBCT with a waiting list control condition. Intervention groups receive treatment immediately; the waiting list control group receives treatment three months later. Both treatments are individually delivered in 8 sessions of 45 to 60 minutes by trained therapists. Primary outcome is severity of depressive symptoms. Anxiety, well-being, diabetes-related distress, HbA1c levels, and intersession changes in mood are assessed as secondary outcomes. Assessments are held at pre-treatment, several time points during treatment, at post-treatment, and at 3-months and 9-months follow-up. The study has been approved by a medical ethical committee.

**Discussion:**

Both CBT and MBCT are expected to help improve depressive symptoms in patients with diabetes. If MBCT is at least equally effective as CBT, MBCT can be established as an alternative approach to CBT for treating depressive symptoms in patients with diabetes. By analyzing moderators and mediators of change, more information can be gathered for whom and why CBT and MBCT are effective.

**Trial registration:**

Clinical Trials NCT01630512.

## Background

Depression is a common comorbidity of diabetes, negatively affecting adherence to medication, dietary and exercise recommendations, and patients’ medical outcomes (Ciechanowski et al., 2000; Ciechanowski et al., 2003). Psychological therapies can be considered as the treatment of choice for depression in somatic patient populations, as they do not interfere with medical treatment regimes, have no physical side-effects, and are often preferred by patients in comparison to antidepressant drugs (Dwight-Johnson et al., 2000; Lustman & Clouse 2002). Additionally, one of the important advantages of a psychological approach over a pharmacological approach to treat depression is that psychological therapies provide patients with tools which enable them to cope with future symptoms of depression, and thereby may reduce the risk of relapse of this highly recurrent disorder. Although there is evidence from systematic reviews and meta-analyses for the efficacy of psychological treatment for depression in patients with diabetes in general (Baumeister et al., 2012; van der Feltz-Cornelis et al., 2010), little is known about which specific types of psychological intervention are effective.

The most commonly used and recommended type of psychotherapeutic intervention for depression is Cognitive Behavioral Therapy (CBT), a short-term intervention focusing on behavioral activation and changing negative thoughts. In a recent meta-analysis on the effectiveness of CBT for depression in patients with a diversity of somatic diseases (including diabetes), CBT was found to significantly reduce depressive symptoms compared to control conditions (Beltman et al., 2010). Specifically in patients with diabetes, only four randomized controlled trials have been conducted so far to test the effectiveness of CBT in treating depression. All four RCTs have found that CBT is effective in reducing depressive symptoms (Lamers et al., 2010; Lustman et al., 1998; Penckofer et al., 2012; van Bastelaar et al., 2011).

In the past decades, another type of cognitive therapy, namely Mindfulness-Based Cognitive Therapy (MBCT) has become increasingly popular, both in clinical practice and research. MBCT integrates CBT with mindfulness. The concept mindfulness has been defined as “paying attention in a particular way: on purpose, in the present moment, and nonjudgmentally” (Kabat-Zinn 2003). Mindfulness-based interventions involve practicing this form of attentiveness, or awareness, both in formal exercises (like meditation and yoga) and in informal exercises in daily life.

CBT and MBCT both encourage awareness of thoughts and feelings and to adequately regulate them, yet they differ in how to learn to adjust to such experiences. In CBT the main components are behavioral activation and critically challenging and replacing the content of negative thoughts, while the main component in MBCT is to learn to relate differently to thoughts and feelings, in a nonjudgmental and accepting way, merely observing them as they come and go. MBCT has been designed as a method to prevent recurrence of depression in patients with prior history of depressive disorder (Segal et al., 2002), yet, there is increasing evidence that MBCT is also effective in the treatment of current depressive symptoms (Hofmann et al., 2010). Among patients with diabetes, only five studies (two observational trials and three randomized controlled trials) have been conducted so far testing the effectiveness of mindfulness-based interventions, showing decreases in psychological distress (Hartmann et al., 2012; Rosenzweig et al., 2007; Schroevers et al., 2013; van Son et al., 2013; Young et al., 2009). A recent randomized controlled trial investigating the effect of MBCT for patients with diabetes found a greater reduction of depressive symptoms in the mindfulness group compared to the waiting list control condition (van Son et al., 2013). Taken the positive effects of MBCT into account, it is now strongly advocated to use a more rigorous design to test its effects, namely by including not only a control group (such as treatment-as-usual or waiting list control), but also an active evidence-based treatment condition, like CBT (Hofmann et al., 2010). To date only a few small trials have directly compared CBT and MBCT as two active treatments (Manicavasagar et al., 2012; Zautra et al., 2008). None of them have been conducted in a diabetic population with depressive symptoms.

### Current study

The present Mood Enhancement Therapy Intervention Study (METIS) aims to study the effectiveness of CBT and MBCT in relation to a control condition. Taking into account the limited number of RCTs and thus limited empirical evidence for the effectiveness of CBT and MBCT for depressive symptoms in patients with diabetes, we believe that it is important to compare both interventions to a control condition. By examining both CBT and MBCT in one trial, our research not only enables the study of their differential effectiveness but it also provides the opportunity to clarify the mechanisms whereby CBT and MBCT are efficacious and to identify for whom each treatment is likely to be most beneficial. Therefore, we explore the role of several mediators, moderators, common factors, and treatment integrity in treatment outcomes, which will be described in more detail in the following part.

Our primary interest is the examination of effects of CBT and MBCT on depressive symptoms in patients with diabetes. In order to study possible effects of a wider range of outcomes, anxiety, well-being, diabetes-related distress, and intersession changes in mood are assessed as secondary outcomes. In addition to investigating secondary effects of CBT and MBCT on these psychological outcomes, we assess effects on the medical outcome HbA1c. Previous studies are inconsistent regarding whether depression treatment also leads to an improvement in diabetes self-management and subsequent glycemic control (Detweiler-Bedell et al., 2008). Two recent RCTs on CBT and MBCT for depression in patients with diabetes found that glycemic control did not improve after psychological treatment (van Bastelaar et al., 2011; van Son et al., 2013) To further clarify this important topic, glycemic control as indicated by HbA1c levels is included as an exploratory secondary outcome.

In this study, both interventions are administered individually in eight face-to-face sessions. Individual CBT for depression has already proven to be effective in persons with a somatic disease, even more than group delivery of CBT (Beltman et al., 2010). In contrast, MBCT is usually delivered and tested in a group setting. Such a group setting may be supportive (Griffiths et al., 2009). However, it has also been indicated that a large group of people prefers individual over group delivery of MBCT (Lau et al., 2012) and that some people participating in a group mindfulness-based intervention found group sharing frustrating and not beneficial (Griffiths et al., 2009). Moreover, it may not always be possible to offer a group program, especially in hospital settings. Group interventions require that all patients are able to come at a common time and patients may have to wait quite a while until a sufficient number of group participants is available. For these reasons, we undertook the challenge to adapt the standardized treatment protocol of group MBCT for individual therapy. A pilot study that investigated the feasibility and acceptability of individual MBCT for people with diabetes and comorbid psychological distress found that most patients were satisfied with the treatment and considered it as helpful. Moreover, MBCT led to reductions in depressive symptoms and diabetes related distress (Schroevers et al., 2013).

#### Mediators

Only recently, research has started to examine why and how MBCT may work in reducing psychological symptoms, by studying underlying mechanisms of change. This research is still in its infancy, especially compared to CBT for which more evidence is available regarding its mediators of effects. Moreover, little is known about the extent to which mechanisms of change are unique or possibly overlapping between CBT and MBCT (Driessen & Hollon, 2010; Shapiro et al., 2006). In order to fill this gap and address this fundamental issue, we investigate mediators of CBT and MBCT and make comparisons among both treatments. Based on previous empirical studies as well as the treatment components and theories underlying CBT and MBCT, three groups of mediators are selected: mediators specific for CBT (e.g., behavioral activation and cognitive reappraisal), mediators specific for MBCT (e.g., mindfulness and self-compassion), and mediators assumed to play a role in both treatments (e.g., overidentification).

#### Moderators

Currently, there is also a lack of information regarding factors that may moderate the effectiveness of treatment. Such information is of clinical importance as it may indicate groups of persons likely or not to benefit from treatment. In order to examine for whom CBT and MBCT are (not) effective, several moderators are examined in the current study. First, we examine the moderating role of baseline psychological factors, including demographic and personality trait factors (i.e. neuroticism, attachment style) (see Bagby et al., 2008; Cordon et al., 2009; McBride et al., 2006) and history of depression, as the latter has been found to play a moderating role in previous MBCT studies (Segal et al., 2002). Second, we explore baseline medical and biological moderating factors. We examine the influence of diabetes specific characteristics (i.e. type and duration of diabetes, diabetes complications, and previous hospital admissions due to severe hypoglycemia) and degradation of tryptophan on treatment outcome. Tryptophan serves as a precursor for serotonin and plays an important role in depression (Russo et al., 2009). Functioning as a natural antidepressant, tryptophan could therefore influence treatment outcome (Thomson, 1982).

#### Common factors

Common factors that are shared by different treatment modalities such as the therapeutic alliance and patients’ treatment expectancies, have been shown to predict positive change in psychotherapy (Martin et al., 2000; Noble et al., 2001). Yet, the role of these factors on treatment outcome has hardly been studied in MBCT. We therefore study the associations between the alliance, expectancies and subsequent change in depressive symptoms among the two treatment conditions.

#### Treatment integrity

It has been firmly recommended to measure treatment integrity in randomized controlled trials to be able to draw valid conclusions on the treatment effects (e.g. Moncher & Prinz, 1991; Perepletchikova et al., 2007). Treatment integrity refers to whether the intervention was implemented as intended (Kazdin & Nock, 2003). Two aspects of treatment integrity are investigated: the extent to which therapists adhere to procedures described in the protocol (treatment adherence) and whether CBT and MBCT differ in the intended manner (treatment differentiation) (Waltz et al., 1993).

Homework assignments are a central component of CBT and MBCT. Research has shown that compliance to homework assignments positively predicts treatment outcome in CBT (Kazantzis et al., 2000). Furthermore, homework compliance may explain the associations between common factors and depressive symptom change (e.g. Westra et al., 2007). In the present study, we examine patients’ compliance to homework as well.

### Study aim and hypotheses

The primary objective of this study is to assess immediate and long-term effects of CBT and MBCT in reducing depressive symptoms in patients with diabetes. We hypothesize that both active treatments are more effective than a waiting list control condition in reducing depressive symptoms. We do not expect CBT or MBCT to be superior over the other. Secondary objectives are to examine potential factors that mediate and moderate treatment effects of MBCT and CBT, as an effort to gain more clarity on why and for whom CBT and MBCT are (not) effective. In addition, we aim to investigate the associations between common factors, treatment integrity, and depressive symptom improvement.

## Methods/design

### Study design

The present study is a multi-center, randomized controlled trial (RCT). Participants are assigned to MBCT, CBT, or a waiting list control condition. Patients allocated to the control group are randomized for the second time and receive one of the two treatments three months later. The choice for a waiting list as a control condition is based on ethical reasons, as patients are screened and all had elevated levels of depressive symptoms. This is also the reason why we chose a waiting period of no longer than three months. Treatment effects are monitored over a period of one year from baseline. This study is conducted in accordance with the principles of the Declaration of Helsinki (version 2008) and the Medical Research Involving Human Subjects Act (WMO) and is approved by the Medical Ethical Committee of the University Medical Center Groningen (UMCG).

### Recruitment and screening procedure

**Figure 1:**
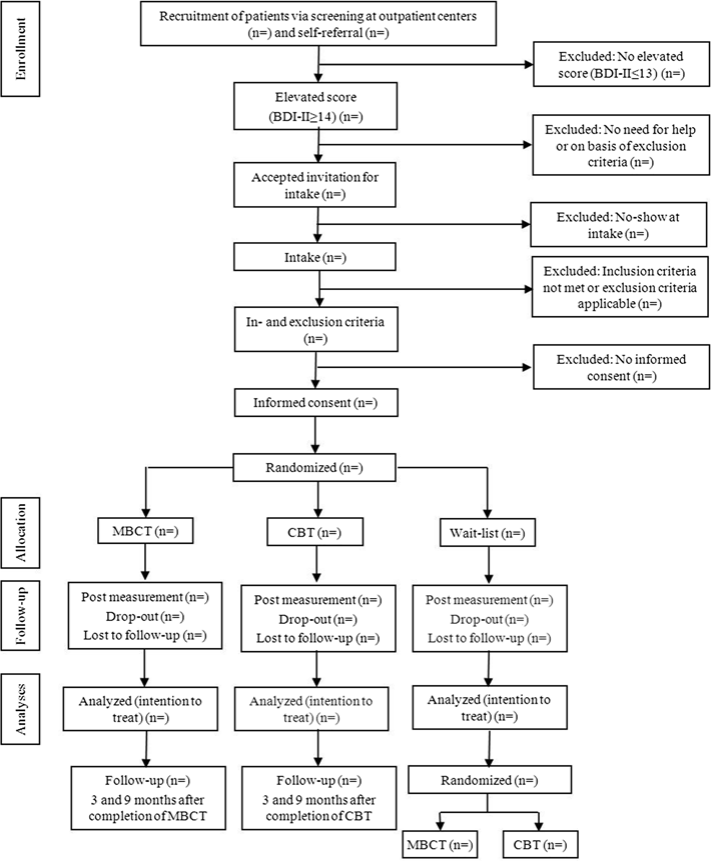
**Participant recruitment and flow through the study.**

Figure 1 illustrates participant recruitment and flow through the study. Patients who are currently receiving medical treatment at one of the participating hospitals for their diabetes are routinely screened for depressive symptoms. They receive a letter from their diabetes outpatient clinic with a request to fill in a questionnaire concerning their mood (Beck Depression Inventory–II (BDI-II) and Well-being Index (WHO-5)), either online or in pen-and-paper version. Several hospitals, primarily in the Northern part of the Netherlands are approached to participate. At present, the University Medical Center Groningen, the Martini Hospital Groningen, the Medical Center Leeuwarden, and the Hospital Rivierenland Tiel have agreed to take part in the study. Patients are also able to participate through self-referral. Patients, whose score on depressive symptoms on the BDI-II is ≥ 14, are invited to meet with one of the psychologists or research assistants involved in the study. Patients who fulfill the inclusion criteria and who give written informed consent for participation are included in the study.

### Study population

*Inclusion criteria are*: diagnosed with type 1 or 2 diabetes at least three months prior to inclusion; ≥ 18 and ≤70 years of age; having depressive symptoms as assessed by the BDI-II score ≥ 14 (cut-off score indicating the presence of at least mild symptoms of depression).

*Exclusion criteria are*: Not being able to read and write Dutch; pregnancy; severe psychiatric comorbidity (i.e., recently experienced psychosis, bipolar disorder, panic disorder, diagnosis of schizophrenia, serious cognitive or neurological problems); acute suicidal ideations or behavior; receiving an alternative psychological treatment during or less than two months prior to starting the participation in the study. Using an antidepressant drug during participation in the present study is allowed, on condition that a patient has been on stable medication regimen for at least two months prior to inclusion in the study, and that no new treatment with an antidepressant is initiated during the course of the study.

### Treatment allocation

Computerized randomization within each hospital is carried out, with participants being stratified by gender, use of antidepressant medication, and their score on the BDI-II at baseline.

### Blinding

Prior to their randomization, patients are blinded to the study condition. No specific information about study design or the type of intervention is given, other than that both treatments focus on learning to cope with negative thoughts or feelings in a different way. The patients are told that the treatment starts relatively soon after they have given their consent, and that a possible waiting period is no longer than three months. After randomization the patients are given more precise information about the treatment they are going to receive.

### Interventions

For the current study, we chose to offer patients with diabetes a generic treatment for depressive symptoms (CBT or MBCT). Our argument for this decision is that depressive symptoms may be related to diabetes for some patients to some extent, but not necessarily for all patients. By offering a generic rather than a diabetes-specific treatment for depressive symptoms, we aim to reduce depressive symptoms in diabetes patients, irrespective of the cause of depressive symptoms and the extent to which they are related to diabetes and/or other important domains in the patients’ lives. During the treatment (CBT or MBCT) patients could bring in diabetes-related topics if relevant for their depressive mood. This way, patients could use the learned CBT or MBCT strategies for managing diabetes-related depressive symptoms.

The treatments consist of eight individual sessions, which are scheduled weekly and last 45 to 60 minutes. Patients receive a workbook with homework assignments and are expected to spend about 30 minutes per day on these assignments. Patients in the MBCT condition also receive audio CDs with mindfulness exercises.

Both CBT and MBCT are led by trained therapists who receive supervision. One MBCT therapist is a diabetes nurse who is a qualified mindfulness therapist, all other therapists have a master’s degree in clinical psychology and most of them have experience with diabetic patients. All therapists have experience in the delivery of the specific treatment (CBT or MBCT) that they are giving to patients. Before start of the study, the therapists receive an additional three day training by an experienced, qualified MBCT or CBT therapist who also provides supervision every three weeks throughout the intervention and study period.

#### CBT

The CBT treatment protocol is based on CBT for depression developed by Beck et al. (1979). The first part of the treatment is devoted to behavioral components of CBT, such as planning and undertaking of (pleasant or functional) activities. The second part of the treatment focuses on dysfunctional thinking patterns, allowing patients to recognize, challenge, and adjust their negative automatic thoughts.

#### MBCT

The MBCT treatment protocol is based on the protocol as developed by Segal et al. (2002). MBCT integrates cognitive therapy for depression with mindful meditation and was originally developed to teach formerly depressed patients new skills in order to help them prevent relapse. Key themes include experiential learning and the development of an open and acceptant mode of (negative) feelings, thoughts, and body sensations (Segal et al., 2002). Formulation of specific prevention strategies is included in a later stage of treatment. Originally, MBCT is given as a 2.5-hour per session group treatment. We developed a shortened and individualized version of this protocol which has previously been tested in a pilot study in patients with diabetes (Schroevers et al., 2013).

### Outcome assessment

Table 1 presents an overview of the measures and the time points on which they are assessed. Assessments take place after consent to participate and before randomization (T1), after the first treatment session (T2), after the second treatment session (T3), after the fourth treatment session (T4), directly after completion of treatment (T5), 3 months after treatment (T6), and 9 months after treatment (T7). The measurements during the course of treatment are used to assess patients’ expectancies, the development of therapeutic relationship, and the process of change in mediator and outcome variables. At T1 and T5, patients are also interviewed and their depressive symptoms are rated by the interviewer by means of a structured clinical interview. The T5 interview also evaluates patients’ acceptability and satisfaction with the received treatment, what they learned, and what they found most or least helpful. All treatment sessions of patients that provide consent are videotaped. Patients are also asked consent for sampling a maximum of 3 ml extra blood in order to assess tryptophan. Participants in the waiting list condition receive the first assessment (T1) twice, first directly after given consent and then again at the end of the 3-months waiting period. After a second randomization, they receive the same assessments as participants in the CBT and MBCT condition. We realize it may be a burden for patients to fill out all these assessments. Therefore, we made careful consideration which questionnaires to include. In order to enhance patients’ commitment to the study and to reduce attrition, all patients are assigned to a specific contact person throughout the study period.

**Table 1:** **Instruments to assess primary outcome, secondary outcomes, moderators, mediators, and common factors**

Concept	Measurement (items)**	Measurement in time points*						
		T1	T2	T3	T4	T5	T6	T7
***Primary outcome measure***								
Depressive symptoms	BDI-II (21)	x		x	x	x	x	x
	HAMD-7 (7)	x				x		
***Secondary outcome measures***								
Generalized anxiety	GAD-7 (7)	x				x	x	x
Well-being	WHO-5 (5)	x				x	x	x
Diabetes-related distress	PAID (20)	x				x	x	x
Intersession changes in mood***	Emotion Thermometers (5)							
Glycemic control (from patients’ records)	HbA1c	x				x		
***Moderators***								
*Psychological moderators*								
Neuroticism	NEO-FFI (12)	x						
Attachment style	ECR-S (12)	x				x		
History of depression	SCID	x						
Demographic characteristics	-	x						
*Biological moderators*								
Diabetes specific characteristics (from patients’ records)	-	x						
Degradation of tryptophan	trp/kyn	x						
***Mediators***								
*Cognitive coping*								
Positive re-interpretation and positive refocusing	CERQ (8)	x			x	x	x	x
Reappraisal and distraction	TCQ (12)	x			x	x	x	x
*Behavioral activation*								
Behavioral activation	BADS (7)	x			x	x	x	x
*Rumination*								
Rumination	RRQ (12)	x			x	x	x	x
*Mindfulness*								
Non-judgmental attitude and act with awareness	FFMQ (16)	x			x	x	x	x
Attention control	Self-Regulation Scale (10)	x			x	x	x	x
*Self-compassion*								
Self-kindness, self-judgment, and overidentification	SCS (13)	x			x	x	x	x
***Common factors***								
Therapeutic alliance	WAI-12 (12) & 18-item Rapport Quest. (18)			x	x			
Patient expectancy	- (3)		x					

* T1 baseline; T2 after first session; T3 after second session; T4 after fourth session; T5 after eighth session (post-treatment); T6 three months after training; T7 nine months after training.

** BDI-II - Beck Depression Inventory-II; HAMD-7 - Hamilton Depression Rating Scale; Emotion Thermometers; GAD-7 - Generalized Anxiety Disorder Scale; WHO-5 Well-being Index; PAID - The Problem Areas in Diabetes scale; NEO-FFI - NEO- Five Factor Inventory; ECR-S - The experiences in close relationship scale short form; SCID - Structured Clinical Interview for DSM-IV; CERQ - Cognitive Emotion Regulation Questionnaire; TCQ - Thought Control Questionnaire; BADS -Behavioral Activation for Depression Scale; RRQ - Rumination-Reflection Questionnaire; FFMQ - Five Facet Mindfulness Questionnaire; Self-Regulation Scale; SCS - Self Compassion Scale; WAI-12 - Working Alliance Inventory; 18-item Rapport Questionnaire.

*** assessed at the start of every treatment session.

Note: this table does not cover measures of treatment integrity.

#### Primary outcome measure

The primary outcome of the study is severity of depressive symptoms as assessed with the Beck Depression Inventory-II (BDI-II) (Beck et al., 1996). In addition to the self-report depression measurement, depressive symptoms are assessed in a semi-structured clinical interview using the 7-Item Hamilton Depression Rating scale (HAM-D7) (Mclntyre et al., 2005).

#### Secondary outcome measures

Psychological secondary outcomes are generalized anxiety measured by the Generalized Anxiety Disorder scale (GAD-7) (Spitzer et al., 2006), well-being measured by the Well-being Index (WHO-5) (Bech, 2004), diabetes-related distress measured by the Problem Areas in Diabetes scale (PAID) (Polonsky et al., 1995), and intersession changes in mood assessed by the Emotion Thermometers Tool (ETT) (Mitchell et al., 2010). Medical secondary outcome is glycemic control as indicated with HbA1c values, which are retrieved from patients’ records. We access the standard measurements of the outpatient clinics and use the average of values half a year before intervention as pre-treatment measure and the values half a year after intervention as post-treatment measure.

#### Moderating factors

The NEO- Five Factor Inventory (NEO-FFI) (McCrae & Costa, 2004) and the Experiences in Close Relationship Scale short form (ECR-S) (Wei et al., 2007) are included as measures of neuroticism and attachment style respectively. History of depression is assessed by the use of the Structured Clinical Interview for DSM-IV (SCID-I) (First et al., 2002). Demographic and diabetes specific characteristics are retrieved from patients’ medical records and patients’ blood samples are used to investigate degradation of tryptophan.

#### Mediating factors

Cognitive coping is measured by two subscales of the Cognitive Emotion Regulation Questionnaire (CERQ), namely positive re-interpretation and positive refocusing (Garnefski & Kraaij, 2007) and by two subscales of the Thought Control Questionnaire (TCQ), namely reappraisal and distraction (Wells & Davies, 1994). Furthermore, to measure behavioral activation we use the Behavioral Activation for Depression Scale (BADS) (Kanter et al., 2007) and to measure rumination we use the Rumination-Reflection Questionnaire (RRQ) (Trapnell & Campbell, 1999). Mindfulness is assessed with two subscales of the Five Facet Mindfulness Questionnaire (FFMQ), namely non-judgmental attitude and act with awareness (Baer et al., 2006). Attention control is assessed with the Self-Regulation Scale (Brown et al., 1999). Self-compassion is measured using three subscales of the Self Compassion Scale (SCS), namely self-kindness, self-judgment, and overidentification (Neff, 2003).

#### Common factors

The Working Alliance Inventory (WAI-12) (Horvath & Greenberg, 1989) and the 18-item Rapport Questionnair*e* (Bernieri, 2005) are selected as measures of patients’ reports of the therapeutic alliance. Patients’ expectancies of improvement are assessed with a three-item credibility questionnaire based on the work of Borkovec and Nau (1972).

#### Treatment integrity

Videotaped treatment sessions are rated on therapists’ adherence to the treatment protocol by two independent observers. Patients’ homework compliance is assessed with checklists that capture the homework assignments proscribed by the MBCT and CBT treatment protocols. Patients are asked to complete the checklist every day of the coming week and to return the checklist at the next therapy session.

### Sample size

The sample size calculation is based on differences in post-treatment depressive symptoms between the waiting list control group and one of the psychological intervention groups. A 5 point difference on the BDI-II (assuming a standard deviation of 8 points) between the waiting list control group and one of the intervention groups is considered a clinically relevant difference. In accordance with previous research (Keers et al., 2005), a Number Needed to Treat of 2.0 is considered cost-efficient and clinical relevant. Stated differently, at least half of participants should improve 5 points on the BDI-II. Testing two-sided, a sample size of 42 patients per group (126 patients in total) yields to an effect size of 0.6 according to Cohen with a power of 80%, and an alpha of 0.05 (Cohen, 2003). This number allows us to test the effectiveness of both interventions, compared to the waiting list control, using intention-to-treat analyses. Allowing a drop-out rate of 25%, we are able to include 32 patients in each of our three conditions in completer analyses.

### Analysis plan

Primary analyses are conducted according to the intention-to-treat approach. To answer the primary research question, repeated measures analyses of (co)variance ((M)AN(C)OVA) are performed, using primary and secondary outcomes as dependent variables and type of treatment as independent variable. If there are significant differences between the patient populations of the different hospitals, we will perform multi-level analyses. Baseline values of dependent variables are included as covariate along with other baseline measures of demographic characteristics that contribute significantly to analysis. Clinical effectiveness is calculated with the reliable change index (RCI) (Jacobson & Truax, 1991). Analyses for mediation and moderation effects, common factors, and treatment integrity are done using condition process analyses in SPSS, growth curve analyses (structural equation modeling), and repeated measures analyses.

## Discussion

Our study is the first to investigate the effectiveness of CBT and MBCT in one randomized controlled trial in patients with diabetes. Both interventions are expected to improve depressive symptoms in patients diagnosed with diabetes and suffering from comorbid symptoms of depression, in comparison to a waitlist control group. In case of positive findings, CBT and/or MBCT may be considered a valuable addition to standard care of patients with diabetes and comorbid depressive symptoms. For ethical reasons, we include a waiting list control condition (rather than treatment-as-usual, TAU) as all participants have elevated levels of depressive symptoms at start of the study. Consequently, we cannot examine long-term effects of CBT and MBCT compared to control condition.

The primary outcome of this study is severity of depressive symptoms, yet we also examine possible effects on several other psychological outcomes as well as on patients’ medical outcome, specifically on values of HbA1c. In order to burden the patients as little as possible, the values are obtained from their medical records instead of scheduling additional measurements at designated time points. A limitation to this approach is that the values are rather general and that we cannot compare CBT and MBCT with the control condition regarding changes in HbA1c values. Yet, we examine pre- to post-treatment changes in HbA1c values for all participating patients.

If MBCT proves to be effective in reducing depressive symptoms in our study, it can be established as a sound alternative to CBT for treating depressive symptoms in patients with diabetes. The choice to study MBCT in an individual therapy mode is novel and may be promising, as not all patients are able and willing to participate in a standard MBCT group treatment. Since both interventions in this study are offered as a structured individualized protocol of eight sessions, they may be especially suitable for a medical setting where patients often receive short individual treatments.

In addition to studying the effectiveness of the two treatment protocols, the current study intends to examine potential unique and joint factors which moderate and mediate treatment effects in CBT and MBCT, and to investigate the associations between common factors, treatment integrity, and depressive symptom improvement. Posing such questions aims for more than just a comparison of treatments in a most straightforward way (do they work or not?), but rather a look under the surface (how, why, and for which patients they might or might not work). A strength of the current study is that we assess predictor variables not only before and after treatment, but also during treatment. This gives us the possibility to measure temporal precedence and make inferences about causality.

By increasing the empirical evidence for the psychological treatment of depressive symptoms in people with diabetes, we hope that insights into which treatment works best for whom and how, will help improve the care of patients with diabetes who experience depressive symptoms.
